# Swimming exercise stimulates IGF1/ PI3K/Akt and AMPK/SIRT1/PGC1α survival signaling to suppress apoptosis and inflammation in aging hippocampus

**DOI:** 10.18632/aging.103046

**Published:** 2020-04-22

**Authors:** Jing-Ying Lin, Wei-Wen Kuo, Rathinasamy Baskaran, Chia-Hua Kuo, Yun-An Chen, William Shao-Tsu Chen, Tsung-Jung Ho, Cecilia Hsuan Day, B. Mahalakshmi, Chih-Yang Huang

**Affiliations:** 1 Department of Medical Imaging and Radiological Science, Central Taiwan University of Science and Technology, Taichung, Taiwan; 2 Department of Biological Science and Technology, China Medical University, Taichung, Taiwan; 3 Department of Bioinformatics and Medical Engineering, Asia University, Taichung, Taiwan; 4 Laboratory of Exercise Biochemistry, University of Taipei, Taipei, Taiwan; 5 Division of Addictive Medicine, Hualien Tzu Chi Hospital, Buddhist Tzu Chi Medical Foundation, Tzu Chi University, Hualien, Taiwan; 6 Department of Chinese Medicine, Hualien Tzu Chi Hospital, Buddhist Tzu Chi Medical Foundation, Tzu Chi University, Hualien, Taiwan; 7 Department of Nursing, MeiHo University, Pingtung, Taiwan; 8 Institute of Research and Development, Duy Tan University, Da Nang, Vietnam; 9 Graduate Institute of Biomedical Sciences, China Medical University, Taichung, Taiwan; 10 Center of General Education, Buddhist Tzu Chi Medical Foundation, Tzu Chi University of Science and Technology, Hualien, Taiwan; 11 Department of Medical Research, China Medical University Hospital, China Medical University, Taichung, Taiwan; 12 Department of Biotechnology, Asia University, Taichung, Taiwan

**Keywords:** swimming exercise, induced-aging rats, hippocampus, inflammatory, apoptosis

## Abstract

Hippocampus is one of the most vulnerable brain regions in terms of age-related pathological change. Exercise is presumed to delay the aging process and promote health because it seems to improve the function of most of the aging mechanisms. The purpose of this study is to evaluate the effects of swimming exercise training on brain inflammation, apoptotic and survival pathways in the hippocampus of D-galactose-induced aging in SD rats. The rats were allocated to the following groups: (1) control; (2) swimming exercise; (3) induced-aging by injecting D-galactose; (4) induced-aging rats with swimming exercise. The longevity-related AMPK/SIRT1/PGC-1α signaling pathway and brain IGF1/PI3K/Akt survival pathway were significantly reduced in D-galactose-induced aging group compared to non-aging control group and increased after exercise training. The inflammation pathway markers were over-expressed in induced-aging hippocampus, exercise significantly inhibited the inflammatory signaling activity. Fas-dependent and mitochondrial-dependent apoptotic pathways were significantly increased in the induced-aging group relative to the control group whereas they were decreased in the aging-exercise group. This study demonstrated that swimming exercise not only reduced aging-induced brain apoptosis and inflammatory signaling activity, but also enhanced the survival pathways in the hippocampus, which provides one of the new beneficial effects for exercise training in aging brain.

## INTRODUCTION

Aging is a gradual process, the accumulation of molecular and cellular damage throughout life leads to a wide range of age-related disorders such as cardiovascular dysfunction and neurodegenerative diseases. Studies have demonstrated that increased levels of reactive oxygen species (ROS) are involved in the detrimental effect of aging-induced cell damage [1]. Natural aging was experimentally modeled by chronic administration of D-galactose in rodents, as it reacts with amino acids in proteins resulting in formation of advanced glycation end-products(AGEs) binding of AGEs to their receptors causes ROS production and AGEs, which are elevated in aging, neurodegeneration and inflammation [2]. Numerous studies indicated that physical exercise exerts neuroprotective effects and increases cognitive activity [3], ameliorates neurological impairments in several neurodegenerative diseases [4] and reduces age-related neuronal loss [5]. Physical exercise is known to have positive effects on the central nervous system via a number of mechanisms, including increases neurotrophic factors production such as VEGF, IGF1, NGF and BDNF that lead to improved efficiency of the vascular system and trigger neuroplastic mechanism in the brain [6–9].

SIRT1, a type III histone/protein deacetylase, is considered a novel anti-aging protein involved in the regulation of variety cell signaling pathways, including apoptosis, cellular senescence, proliferation, oxidative stress, energy metabolism, inflammation and DNA damage response [10]. SIRT1 is abundantly expressed in several regions of the brain, including the hypothalamus, cortex, striatum and hippocampus [11]. A neuroprotective effect of SIRT1 has been reported for both acute and chronic neurological diseases [12]. SIRT1 regulates p53, NF-κB, FOXO family, PGC-1α, and other transcription factors via deacetylation [12]. Caloric restriction and resveratrol intake slow the aging process and promote successful brain aging, with SIRT1 being a key regulator [12]. The AMP-activated protein kinase (AMPK) and SIRT1 are evolutionary conserved partners which have similar functions in metabolism, energy balance and cellular survival [13]. Recently, the effect of exercise on SIRT1 expression and activity has started to be investigated. In aged rats, SIRT1 activity has been reported to increase after a 6 weeks treadmill training program in heart and adipose tissue [14]. In contrast, in rat cerebellum SIRT1 activity was decreased with aging and it was not increased by mild exercise [15]. In humans, three days of cycling caused increases in mRNA of SIRT1 and PGC-1α in skeletal muscle [16] and marathon running increased SIRT1 and other anti-apoptotic gene expression in peripheral blood mononuclear cells [17]. While most of the effects of exercise on SIRT1-dependent pathways have been described in peripheral tissues, these observations allow hypothesizing that some of the beneficial neurophysiological effects of exercise could be mediated by the modulation of SIRT1 activity in the brain.

Several evidences have indicated that IGF1 plays a crucial role in protection of neurons and low IGF1 levels are associated with brain aging [18]. Activation of IGF1 is beneficial to improve neuronal survival during brain aging [19]. These actions are mediated by IGF1 receptors widely distributed in the brain [20]. The cell survival property of IGF1R signaling is mediated by the activation of the phosphatidylinositol 3-kinase (PI3K) and protein kinase B (Akt) [21, 22]. Phosphorylated Akt serves as a pro-survival signaling pathway by affecting the Bcl-2 family [23]. Bcl-2 family can be divided into pro-apoptotic and pro-survival subgroups. Bcl-2 and Bcl-xL are pro-survival proteins that prevent the activation of downstream apoptotic signaling. However, IGF1R and Bcl-2 family associated pro-survival pathway in aging brain is still not completely understood.

Implicated inflammation was found in aging process and neurodegenerative diseases [24]. It was suggested that alterations in pro-inflammatory cytokines levels, such as tumor-necrosis factor alpha (TNFα), which can potently be induced following brain injury and promote neuroinflammation and neurodegeneration [25]. TNF-α pathway is mediated through two distinct cell surface receptors: TNFR1 and TNFR2. Only TNFR1 contains a cytoplasmic death domain and may directly induce apoptosis [26]. NF-κB also regulates expression of various neurotrophic proteins implicated in neuronal development, function and survival factors [27]. Genes known to be regulated by NF-κB include cyclooxygenase-2 (COX-2) and inducible nitric oxide synthase (iNOS) [27]. Excessive NO generation by iNOS may be harmful to the brain.

Many studies have demonstrated that apoptosis may contribute to the loss of neuronal cells in neuropathy and is regarded as a predictor of adverse outcomes in subjects with age-related neurodegenerative diseases [28]. Apoptosis has two main pathways, extrinsic Fas-dependent and intrinsic mitochondrial-dependent apoptotic pathways. The extrinsic Fas-dependent pathway is initiated by binding the Fas ligand to the Fas receptor. After ligand binding, Fas-receptor oligomerization results in the recruitment of the Fas associated death domain (FADD) adaptor protein and the activation of caspase 8, which is upstream of caspase 3 that is responsible for DNA-cleavage action [28]. Mitochondrial-dependent apoptotic pathway is mediated by internal factors, such as free radicals. In this pathway, mitochondria play an important role in apoptosis by releasing cytochrome c and activating caspase 9, which activates caspase 3. Our recent study indicated that diabetes and exposure of side stream smoke cause Fas receptor-dependent and mitochondrial-dependent apoptosis in animal brains [29, 30].

In this study, we will determine the expression patterns of SIRT1 and related longevity genes in control and induced aging brain of rats, and investigated whether swimming exercise affected their expression. Furthermore, we want to understand whether the IGF1R signaling and pro-survival Bcl-2 family associated pathways in induced- aging brain are worse than control rats and whether these components can be improved by swimming exercise program. We hypothesized that aging may predispose to more impaired neuronal IGF1R/Akt and pro-survival Bcl-2 family pathways, as well as swimming exercise may enhance neuronal IGF1R/Akt survival and AMPK/SIRT1 pathways and prevent apoptotic activity in aging hippocampus.

## RESULTS

### Pathohistological changes of hippocampal neurons

**Figure 1:**
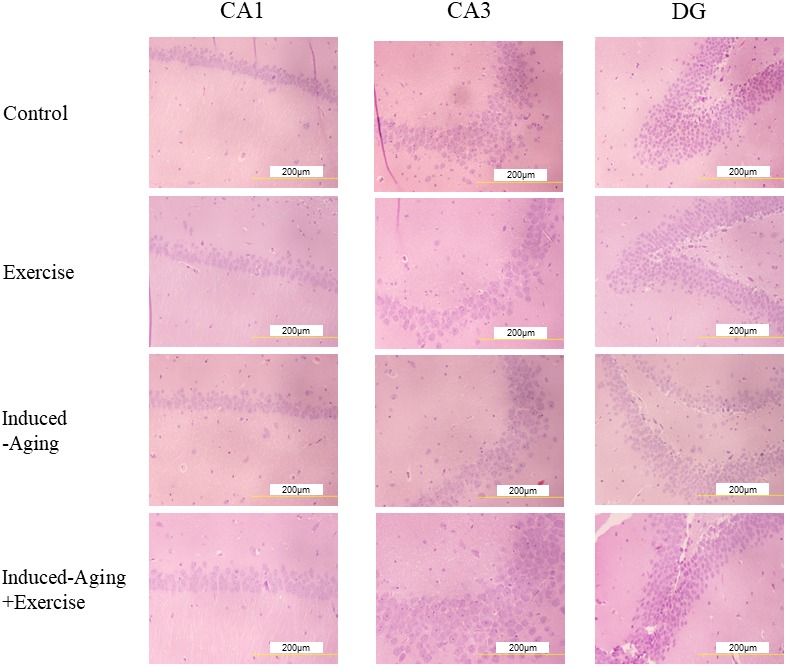
**Representative micrographs of H&E staining in the CA1, CA3 and dentate gyrus (DG) regions of hippocampus for each group.** The images of hippocampus architecture were magnified 400 times.

To investigate changes of neuronal architecture in D-galactose-induced ageing after swimming exercise, we performed a histopathological analysis of hippocampal tissue stained with hematoxylin and eosin (HE). Figure 1 shows representative micrographs of HE staining in the cornu ammonis 1 (CA1), cornu ammonis 3 (CA3) and dentate gyrus (DG) regions of hippocampus for each group. D-galactose-induced aging showed the tendency to decrease the neuronal cell density, which was increased by swimming exercise treatment.

### AMPK/SIRT1 longevity-related signaling pathway

**Figure 2:**
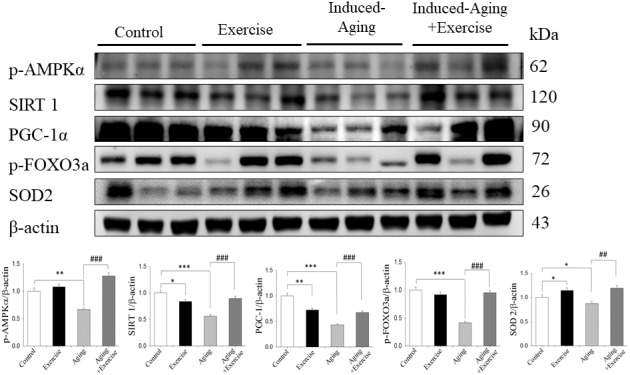
**Exercise training enhanced AMPK/SIRT1 anti-aging pathway in D-galactose-induced aging rat hippocampus.** The representative protein levels of p-AMPKα, SIRT1, PGC-1α, p-FOXO3a, SOD2 prepared from hippocampi in the control, exercise, aging and aging with exercise rats were measured by Western blotting analysis (n=3). The protein expression folds were normalized with β-actin. * *P* <0.05, **P<0.01, ***P<0.001, significant differences from the control group. ^##^ *P* <0.05, ^###^ *P* <0.01, significant differences from the D-galactose-induced aging group.

To identify the effects of D-galactose-induced aging and swimming exercise on the p-AMPK/SIRT1 longevity-related signaling pathway in rat hippocampus, western blotting was conducted. The protein levels of p-AMPK, SIRT1, PGC-1α, p-FOXO3a and SOD2 were significantly decreased in induced-aging group compared with those in the control group. However, these proteins levels in the induced-aging+exercise group were significantly increased compared with the induced-aging group (*p*<0.05, Figure 2). In the control group, swimming exercise mildly enhanced the expression of p-AMPK and SOD2. And swimming exercise mildly suppressed the expression of SIRT1 and PGC-1α (Figure 2).

### IGFI-R/PI3K/Akt survival pathway

**Figure 3:**
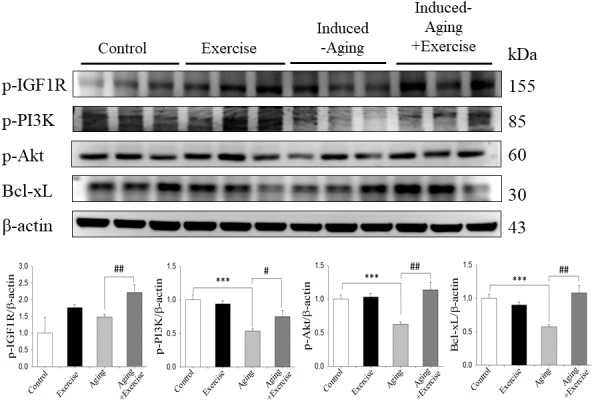
**Exercise training enhanced IGF1/Akt survival pathway in D-galactose-induced aging rat hippocampus.** The representative protein levels of survival proteins p-IGF1 receptor (p-IGF1R), p-PI3K, p-Akt and pro-survival Bcl family of Bcl-xL prepared from hippocampi in the control, exercise, aging and aging with exercise rats were measured by Western blotting analysis (n=3). The protein expression folds were normalized with β-actin. ***P<0.001, significant differences from the control group. ^##^ *P* <0.05, ^###^ *P* <0.01, significant differences from the D-galactose-induced aging group.

To investigate the correlation between longevity-related signaling molecules and the IGF1R/Akt survival pathway, we examined the protein levels of p-IGFIR, p-PI3K, p-Akt and Bcl-xL. The protein levels of p-PI3K, p-Akt and Bcl-xL in the induced-aging group were significantly lower than those in the control group, but p-IGF1-R expression was not altered. However, swimming exercise effectively enhanced the expression of those survival proteins in induced-aging+exercise group compared to induced-aging group (Figure 3). Conversely, swimming exercise alone exerted no significant effect on the expression of these survival proteins compared to control group (Figure 3).

### Stress-related inflammatory proteins

**Figure 4:**
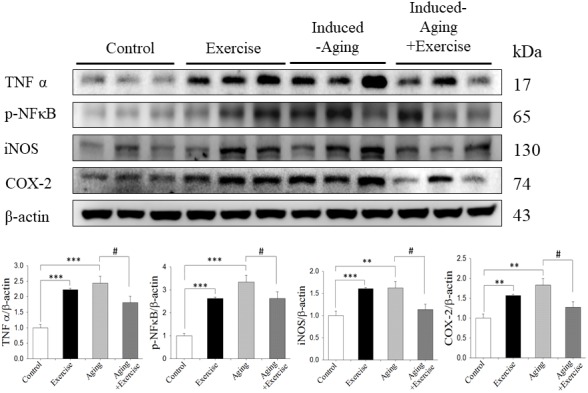
**Effects of exercise training on inflammatory proteins.** The representative protein levels of TNFα, p-NFκB, iNOS and COX2 prepared from hippocampi in the control, exercise, aging and aging with exercise rats were measured by Western blotting analysis (n=3). The protein expression folds were normalized with β-actin. **P<0.01, ***P<0.001, significant differences from the control group. ^##^ *P* <0.05, significant differences from the D-galactose-induced aging group.

To verify the effects of swimming exercise and aging on inflammatory proteins, we examined the protein levels of TNFα, p-NFκB, COX-2 and iNOS for each group. It was observed a marked increase on hippocampal TNFα, p-NFκB, COX-2 and iNOS levels in the induced-aging group when compared to the control group. Swimming exercise attenuated the aging induced increase in the expression of these proteins (Figure 4). Conversely, swimming exercise significantly increased the expression of those inflammatory proteins compared to control group (Figure 4).

### Components of Fas receptor-dependent and mitochondrial-dependent apoptotic pathways

**Figure 5:**
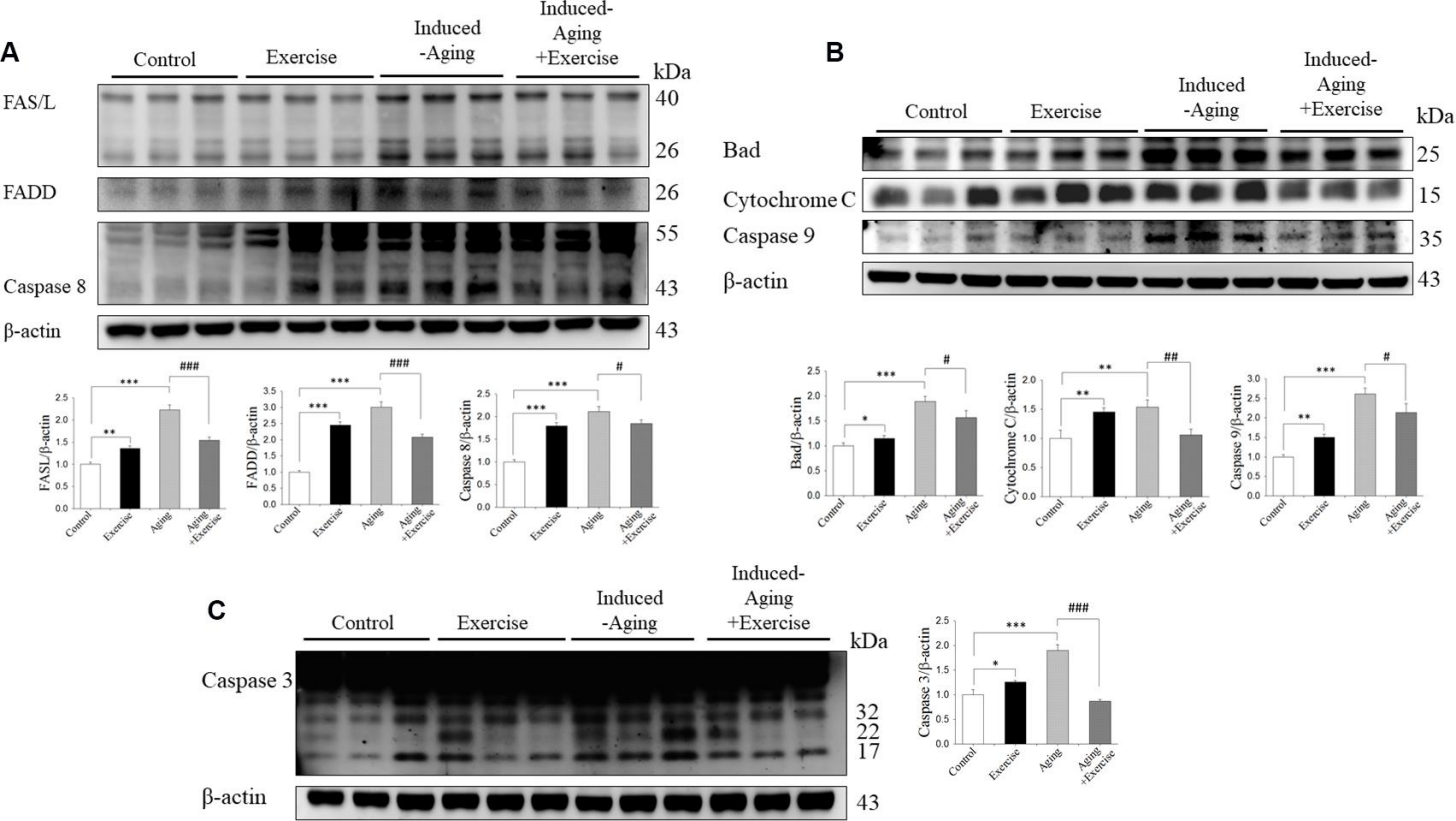
**Exercise training inhibited components of FAS-dependent and mitochondrial-dependent apoptotic pathways activation in hippocampus of D-galactose-induced aging rats.** (**A**) The representative protein levels of FAS ligand, activated FAS receptor, FADD, activated Caspase 8. (**B**) Bad, Cytochrome C, Caspase 9 and (**C**) Caspase 3 prepared from hippocampal homogenates in the control, exercise, aging and aging with exercise rats were measured by Western blotting analysis (n=3). The protein expression folds were normalized with β-actin. * *P* <0.05, **P<0.01, ***P<0.001, significant differences from the control group. ^##^ *P* <0.05, ^###^ *P* <0.01, ^###^*P*<0.001.significant differences from the D-galactose-induced aging group.

**Figure 6:**
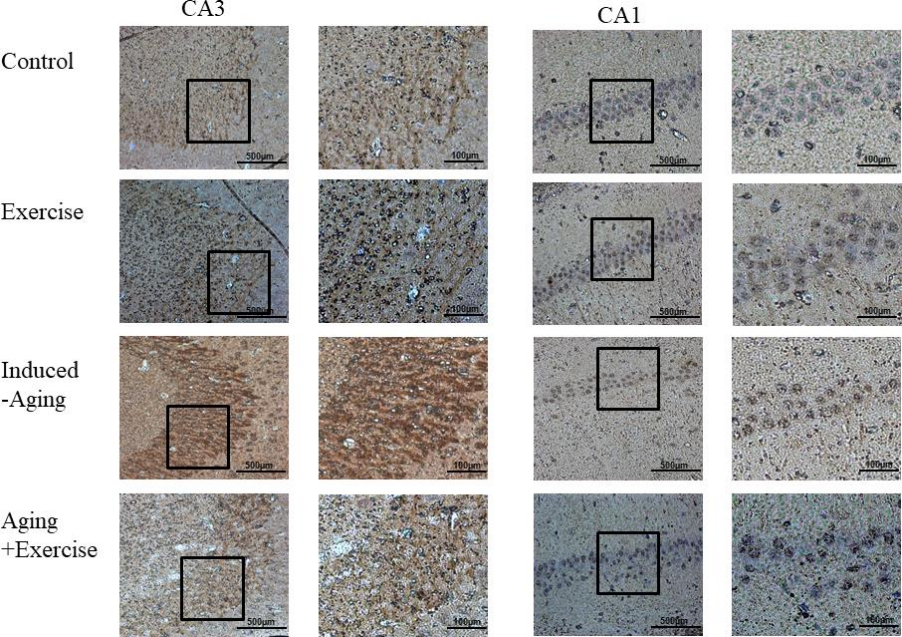
**Photomicrographs of caspase 3-positive cells in the CA1 and CA3 of hippocampus.** The sections were stained for caspase-3 immunoreactivity (brown).

The components of the Fas dependent apoptotic signaling pathways in the hippocampus of rats from control, swimming exercise, induced-aging and induced-aging+ exercise were investigated. We measured the upstream components of the Fas dependent apoptotic pathways including the protein levels of Fas ligand, FADD, pro-caspase 8 and activated caspase 8 from all four groups. Compared with control animals, the protein levels of Fas ligand, FADD and activated caspase 8 significantly increased in the induced-aging group (Figure 5A). In contrast, significantly reduced Fas ligand, FADD and activated caspase 8 were detected in the hippocampus of rats from induced-aging+ exercise group compared to the induced-aging group (Figure 5A). Furthermore, we examined the Bad, cytochrome *c*, activated caspase 9 and activated caspase 3 protein levels to reveal changes in the mitochondrial-dependent apoptotic pathway. The Bad, cytochrome *c*, activated caspase 9 and activated caspase 3 protein levels were significantly higher in induced-aging than in control animals (Figure 5B). Expression of these Fas dependent and mitochondrial-dependent apoptotic protein levels were significantly increased by D-galactose-induced aging. However, swimming exercise in aging-induced significantly reduced these apoptotic protein levels (Figure 5A–5C). In contrast, swimming training alone increased the expression of these Fas dependent and the mitochondrial-dependent apoptotic proteins when compared to the control group. To confirm the apoptosis in induced-aging after swimming exercise, we examined the caspase 3-positive cells in CA3 and CA1 of hippocampus by immunohistochemistry assay. Expression of caspase 3 was significantly increased by D-galactose-induced aging. In contrast, swimming exercise remarkably suppressed caspase 3 expression in the induced-aged group (Figure 6).

## DISCUSSION

**Figure 7:**
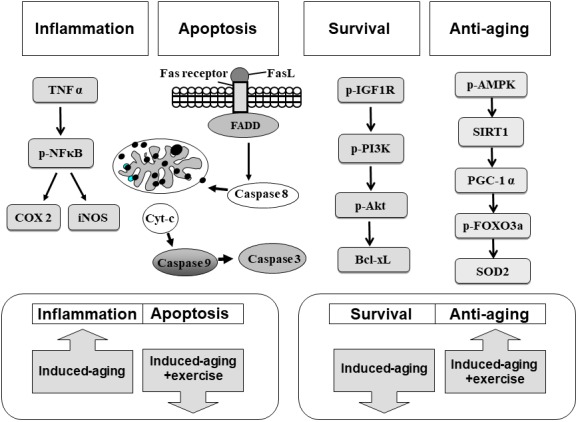
**Our hypothesis that exercise training suppressed inflammatory and apoptotic pathways and enhanced IGF1R/PI3K/Akt survival and anti-aging AMPK/SIRT1/PGC-1α associated pathways in the hippocampus of D-galactose induced aging rats.** These aging attenuated IGF1R related PI3K/Akt survival pathway and AMPK/SIRT1/PGC-1α pathway were improved after exercise training. Activated inflammatory and apoptotic activity were prevented after exercise training.

Our main findings can be summarized as follows (Figure 7): (1) The Fas dependent, mitochondrial-dependent apoptotic pathways and inflammatory proteins exhibited increased levels with a reduced IGFI-R/PI3K/Akt and AMPK/SIRT1 survival pathways in the hippocampus of D-galactose induced aging rats. (2) Swimming exercise not only enhanced IGFI-R/PI3K/Akt and AMPK/SIRT1 survival pathways but also reduced the apoptotic and inflammatory pathways in hippocampus from D-galactose induced aging rats. (3) Swimming exercise modulated the apoptotic and inflammatory pathways in an induced aging-dependent manner, swimming exercise was able to reverse the increases of the apoptotic and inflammatory pathways activity induced by aging process, while increasing the levels of apoptotic and inflammatory protein in control hippocampus.

D-Galactose is a reducing sugar that can be converted into advanced glycation end-products in vivo and in vitro. Excessive D-galactose intakes lead to ROS production in vivo, resulting in chronic inflammation, which facilitates the aging process [31]. In many reports, it was suggested that long-term systemic exposure of D-galactose in rodents causes progressive decline in cognitive function and mimics aging progress, such as hippocampus-dependent cognitive dysfunction, neurodegeneration and impairments in antioxidant capacity [1]. Physical exercise training ameliorates age-related neuronal loss and is currently recommended as a non-pharmacological approach to alleviate symptoms in several neurodegenerative diseases. Although it is clear that SIRT1 and SIRT1-related genes are important in cell survival, their regulation during aging and whether they are part of neuroprotective pathways associated with physical exercise in the central nervous system are unknown. In this study, it was found that SIRT1-related anti-oxidant proteins p-AMPK, SIRT1, PGC-1α, p-FOXO3a and SOD2 in D-galactose-induced aging rats were significantly decreased compared to non-aging control and the impaired SIRT1 anti-oxidant pathway was restored after 8 weeks swimming exercise training. In the aspect of the status of energy metabolism after exercise training, AMPK protein is a crucial regulator of energy metabolic homeostasis at the cellular and whole organism levels and it is a Ser/Thr kinase that is activated upon alterations in the cellular AMP/ATP ratio. In previous studies, AMPK was shown to be increased in human skeletal muscle during exercise [32]. Recently, Marosi et al. also showed enhanced p-AMPK and PGC-1α levels after 15 weeks of exercise training in the hippocampus from aging rats [1]. PGC-1α could be an important modulator of redox balance in neurons, because it controls mitochondrial activity and is required for the induction of the several antioxidant enzymes including SOD-1 and SOD-2 [31, 33]. SIRT1 deacetylates FOXO3 and increases cellular resistance to oxidative stress and inhibits cell death. Overexpression of FOXO3 protects motor neurons from apoptosis induced by mutant SOD1 or polyQ-expanded androgen receptor [34], indicating a neuroprotective role of FOXO3. FOXO3 mRNA expression was negatively correlated with aging in hippocampus and cortex [35]. Therefore, in this study, D-galactose-induced aging hippocampus of brain reduced AMPK/SIRT1 survival and anti-oxidant pathway. Moreover, 8 weeks swimming exercise training enhances this survival pathway.

IGFI-R, PI3K and p-Akt survival proteins and pro-survival protein Bcl-2 in brains of D-galactose-induced aging rats were significantly reduced compared to control. Swimming exercise training facilitated the IGFI-R/PI3K/Akt survival pathway in induced-aging hippocampus. IGF1R and their downstream PI3K and Akt signaling pathways is a critical mediator in exercise-induced physiological cell proliferation, exercise intervention has been shown to reverse functional and molecular abnormalities associated with brain pathology via increased IGF1 or/and PI3K activity [36]. In contrast to the data obtained from the aged group, our swimming exercise protocol did not modify the IGFI-R/PI3K/Akt survival pathways in hippocampus from control rats, showing different profiles between non-aging control and induced-aging rats. This suggests that stimulation of the IGFI-R/PI3K/Akt survival pathways in the hippocampus by this exercise protocol only when aging or disease process in involved. However, D-galactose induced aging does not have effect on p-IGF1-R expression. In our previous study, aging alone did not alter p-IGF1-R expression in heart, however, swimming exercise in aged rats increased p-IGF1-R expression [37].

Inflammatory has been implicated in various pathological conditions involving in aging and was commonly used as an index of brain injury in humans and animals [24]. In this study, D-galactose induced a significant increase of inflammation in the hippocampus, suggesting brain damage caused by D-galactose (Figure 4). Lovatel et al. reported that forced treadmill exercise decreased pro-inflammatory markers, TNFα, IL-1β and NFκB in hippocampus from 20 months old rats [38]. Exercise was able to reduced TNFα and IL-1β levels in transgenic model of Alzheimer Disease [39]. However, in our present study swimming exercise alone induced the inflammatory protein expression level, which might be due to intensity of swimming exercise.

Programmed cell death has a critical role in neuronal cell death during aging process [4, 28]. Many studies have shown that aging can induce excessive apoptosis in the aged rat brain [36, 40]. Studies describing the aging effects on apoptosis focused on the activation of caspase 3. Since an increased number of apoptotic cells was detected in aging rat brain, the apoptotic pathway induced by aging is still unclear. In our study, both the FAS receptor-dependent and mitochondria-dependent apoptotic pathways were activated by D-galactose-induced aging. These two major apoptotic pathways in D-galactose-induced aging were significantly downregulated after two months of swimming exercise training, in which decreases of FAS receptor-dependent apoptotic proteins and mitochondria-dependent apoptotic proteins in the exercise training group compared with the D-galactose-induced aging group (Figure 5).

Exercise training has been known to provide multiple benefits such as anti-hypertension, anti-diabetes, anti-obesity and anti-lipidemia [41]. In this study, non-aging control rats with swimming exercise training also displayed inflammation and apoptosis in the hippocampus. Swimming exercise training modulated the apoptotic and inflammatory pathways in an induced aging-dependent manner, exercise training was able to reverse the increases of the apoptotic and inflammatory pathways activities induced by aging process, while increasing the levels of apoptotic and inflammatory protein in non-aging control rats. Swimming exercise training upregulated apoptosis and inflammation in non-aging control rat; however, higher levels of IGFI-R/PI3K/Akt survival and AMPK/SIRT1 anti-oxidant pathways was able to balance out effects of free radical and ROS. In previous studies, strenuous exercise is accompanied by changes of the expression of numerous cytokines, hormones, growth factors and oxidation-reduction status. All these factors are known to potentially mediate either accelerated death or prolonged survival of leukocytes [42]. Innate and adaptive immunities are major host defense mechanisms which not only can provoke inflammation in order to protect organism against invading pathogens, but also repair tissue injuries and alert the immune system from jeopardizing cellular damage [43]. In another study, intense exercise resulted in apoptosis in the hippocampus through calcium channels [44]. In many reports, it was suggested that modest or aerobic exercises, which exert protective and beneficial effects in pathologic or aging states.

In conclusion, we demonstrated that 8 weeks swimming exercise suppresses inflammation and apoptosis in the hippocampus of induced- aging in male rats by inducing the activation of the AMPK/SITR1-related and IGFI-R/PI3K/Akt survival pathways. Limitation of the present study are: Different extrinsic and intrinsic factors regulate the aging process, which used to differ to tissue types. Comparing the data with natural senescence process could address more reliable molecular mechanism preventive or therapeutic effects of exercise intervention. How prolonged the effect of swimming exercise also needs to be considered.

## MATERIALS AND METHODS

### Animals

The male Sprague-Dawley rats (n=24; 3 month old) were obtained from the National Center for Experimental Animals (National Science Council, Taiwan, Republic of China). All animal were kept in a temperature and light-controlled environment with a 12-hour light/12-hour dark cycle. All protocols were approved by the Institutional Animal Care and Use Committee of Central Taiwan University of Science and Technology, Taichung, Taiwan, and the principles of laboratory animal care (NIH publications) were followed.

Rats were allocated to the following groups: (1) control group (n=6); (2) control rats with swimming exercise group (n=6); (3) induced-aging control group (n=6), aging rats were induced and injected by the intraperitoneal injection of D-galactose (150 mg/kg of body weight, Sigma, St. Louis, MO, USA) which was dissolved in distilled water. D-Galactose was injected at 6.00 P.M. once daily for 8 weeks to induce aging; (4) Induced-aging rats with swimming exercise group (n=6). The exercise rats were made to perform swimming exercise for 5 days per week for 12 weeks. Freestyle swimming was performed, inside a container with a water temperature of 32±2°C. After the swimming exercise was completed, body temperature of the rats were maintained using a hair dryer. The duration of the swimming exercise were gradually increased from 20 min in the first and second weeks, to 30 min in the third week and 60 min from the fourth week onwards [37, 45]. In contrast, rats from the control and aging control groups were placed on the container for the same time points without swimming. To avoid any acute effect of exercise, all animals were sacrificed under deep anesthesia chlorohydrate (400 mg/kg) 48 h after exercise.

### Hematoxylin and eosin (H&E) staining

The hippocampus from brain were excised and soaked in formalin and covered with wax. The 0.2-μm sections were cut from paraffin-embedded tissue blocks. Slides were prepared by daparaffinization. They were passed through a series of graded alcohols (100%, 95% and 75%) for 15 min each. The slides were dried with hematoxylin and eosin. After rinsing with water, each slide was soaked in 85% alcohol, 100% alcohol I and alcohol II for 15 min each. The final step was soaked in xylene I and xylene II. Photomicrographys were obtained using Zeiss Axiophot microscopes.

### Western blotting

Hippocampus was homogenized in ice-cold lysis buffer for 1 min. The homogenates were centrifuged at 12,000 g for 40 min twice. The supernatant was collected and stored at -70°C for further experiments. Protein concentration of tissue extracts was determined by the Bradford method (Bio-Rad Protein Assay, Hercules, CA). Protein homogenates were separated on a 10% SDS-PAGE with a constant voltage of 75 V. Electrophoresed proteins were transferred to polyvinylidene difluoride (PVDF) membrane (Millipore, Bedford, MA, 0.45 μm pore size) with a transfer apparatus (Bio-Rad). PVDF membranes were incubated in 5% non-fat milk in TBS buffer at room temperature for 1hour. Primary antibodies including Fas ligand, Bcl-xL, Bax, Bid, caspase-8, caspase-3, IGF1R, phospho-Akt, acetylated protein, SIRT1, FoxO3a, phospho-FoxO3a (p-FoxO3a, Ser253), (Santa Cruz Biotechnology, Santa Cruz, CA) and α-tubulin (Neo Markers, Fremont, CA) were diluted in antibody binding buffer overnight at 4°C. The blots were washed 3 times in TBS buffer for 10 min and then immersed in the second antibody solution containing goat anti-mouse IgG-HRP, goat anti-rabbit IgG-HRP (Santa Cruz) for 2 h and diluted in TBS buffer. The membranes were washed 3 times for 10 min in TBS buffer. The immunoblotted proteins were visualized using an ECL Western blotting luminal reagent and quantified using a Fujifilm LAS-3000 chemiluminescence detection system (Tokyo, Japan).

### Statistical analysis

SPSS software Version 17 was used for performing statistical analysis. The mean and SE of each treatment group were calculated for all experiments with statistical analysis. The number of samples is indicated in the description of each experiment. All statistical tests were two sided. *P* < 0.05 was considered statistically significant. In all experiments, variance was analyzed by one-way ANOVA followed by Tukey’s multiple comparison.
