# Correction for: Swimming exercise stimulates IGF1/PI3K/Akt and AMPK/SIRT1/PGC1α survival signaling to suppress apoptosis and inflammation in aging hippocampus

**DOI:** 10.18632/aging.104045

**Published:** 2020-08-30

**Authors:** Jing-Ying Lin, Wei-Wen Kuo, Rathinasamy Baskaran, Chia-Hua Kuo, Yun-An Chen, William Shao-Tsu Chen, Tsung-Jung Ho, Cecilia Hsuan Day, B. Mahalakshmi, Chih-Yang Huang

**Affiliations:** 1 Department of Medical Imaging and Radiological Science, Central Taiwan University of Science and Technology, Taichung, Taiwan; 2 Department of Biological Science and Technology, China Medical University, Taichung, Taiwan; 3 Department of Bioinformatics and Medical Engineering, Asia University, Taichung, Taiwan; 4 Laboratory of Exercise Biochemistry, University of Taipei, Taipei, Taiwan; 5 Division of Addictive Medicine, Hualien Tzu Chi Hospital, Buddhist Tzu Chi Medical Foundation, Tzu Chi University, Hualien, Taiwan; 6 Department of Chinese Medicine, Hualien Tzu Chi Hospital, Buddhist Tzu Chi Medical Foundation, Tzu Chi University, Hualien, Taiwan; 7 Department of Nursing, MeiHo University, Pingtung, Taiwan; 8 Institute of Research and Development, Duy Tan University, Da Nang, Vietnam; 9 Graduate Institute of Biomedical Sciences, China Medical University, Taichung, Taiwan; 10 Center of General Education, Buddhist Tzu Chi Medical Foundation, Tzu Chi University of Science and Technology, Hualien, Taiwan; 11 Department of Medical Research, China Medical University Hospital, China Medical University, Taichung, Taiwan; 12 Department of Biotechnology, Asia University, Taichung, Taiwan

**Keywords:** correction

**This article has been corrected:** The authors requested to add missing affiliation of corresponding author Chih-Yang Huang. This correction does not change the content of the publication. The correct list of authors and affiliations is provided below:

Jing-Ying Lin^1,*^, Wei-Wen Kuo^2,*^, Rathinasamy Baskaran^3^, Chia-Hua Kuo^4^, Yun-An Chen^2^, William Shao-Tsu Chen^5^, Tsung-Jung Ho^6^, Cecilia Hsuan Day^7^, B. Mahalakshmi^8^, Chih-Yang Huang^9,10,11,12,13^

^1^Department of Medical Imaging and Radiological Science, Central Taiwan University of Science and Technology, Taichung, Taiwan

^2^Department of Biological Science and Technology, China Medical University, Taichung, Taiwan

^3^Department of Bioinformatics and Medical Engineering, Asia University, Taichung, Taiwan

^4^Laboratory of Exercise Biochemistry, University of Taipei, Taipei, Taiwan

^5^Division of Addictive Medicine, Hualien Tzu Chi Hospital, Buddhist Tzu Chi Medical Foundation, Tzu Chi University, Hualien, Taiwan

^6^Department of Chinese Medicine, Hualien Tzu Chi Hospital, Buddhist Tzu Chi Medical Foundation, Tzu Chi University, Hualien, Taiwan

^7^Department of Nursing, MeiHo University, Pingtung, Taiwan

^8^Institute of Research and Development, Duy Tan University, Da Nang, Vietnam

^9^Graduate Institute of Biomedical Sciences, China Medical University, Taichung, Taiwan

^10^Center of General Education, Buddhist Tzu Chi Medical Foundation, Tzu Chi University of Science and Technology, Hualien, Taiwan

^11^Department of Medical Research, China Medical University Hospital, China Medical University, Taichung, Taiwan

^12^Department of Biotechnology, Asia University, Taichung, Taiwan

^13^Cardiovascular and Mitochondrial Related Disease Research Center, Hualien Tzu Chi Hospital, Buddhist Tzu Chi Medical Foundation, Tzu Chi University of Science and Technology, Hualien, Taiwan

*Equal contribution

Original article: Aging. 2020; 12:6852–6864.  . https://doi.org/10.18632/aging.103046

